# Supplementary material for: Non-Hermitian control between absorption and transparency in perfect zero-reflection magnonics
Source: Nat Commun. 2023 Jun 10;14:3437. doi: 10.1038/s41467-023-39102-3 (PMC10257721; doi:10.1038/s41467-023-39102-3)
Supplement: Supplementary file 1 — Supplementary Information [file 41467_2023_39102_MOESM1_ESM.pdf]

## Supplementary Information

# Non-Hermitian Control between Absorption and Transparency in Perfect Zero-Reflection Magnonics

Jie Qian[1,2], C. H. Meng[1], J.W. Rao[3], Z. J. Rao[1], Zhenghua An[1,4,*], Yongsheng Gui[2] and C. -M. Hu[2,*]

**This PDF file includes:**

**Supplementary Note 1:** Theoretical description of the waveguide magnonic system 2
(1) Effective non-Hermitian Hamiltonian of the system 2
(2) The classical model and spectra 3
(3) Fabry-Pérot (FP)-like resonant mechanism 5

**Supplementary Note 2:** Zero-reflection condition and perfect zero-reflection condition 6
(1) Zero-reflection (ZR) condition 6
(2) Perfect zero-reflection (PZR) condition 6

**Supplementary Note 3:** Experimental spectra of transmission, reflection and absorption 7

**Supplementary Note 4:** Discussion about the maximal energy absorption 9

**Supplementary References** 10

**Supplementary Note 1. Theoretical description of the waveguide magnonic system**

**(1) Effective non-Hermitian Hamiltonian of the system.** Under the rotating-wave approximation, the Hamiltonian of the whole system is,

$$H/\hbar = (\omega_1 - i\gamma_1)\hat{a}^\dagger\hat{a} + (\omega_2 - i\gamma_2)\hat{b}^\dagger\hat{b} + \sum_k \omega_k\, p_k^\dagger p_\mathrm{k} + \sum_k \omega_k\, q_k^\dagger q_\mathrm{k}$$

$$+\sum_k g_1\, a_1^\dagger(p_k + q_k) + \sum_k g_2\, e^{i\Phi} a_2^\dagger(p_k + q_k) + h.c. \qquad \text{(S1)}$$

where $\hat{a}^\dagger(\hat{a})$ and $\hat{b}^\dagger(\hat{b})$ represent the creation (annihilation) operators of the two magnon modes, $\omega_{1,2} - i\gamma_{1,2}$ are their complex frequencies, with the real and imaginary parts representing the resonant frequency and intrinsic damping rates, respectively. $p_k^\dagger(p_\mathrm{k})$ and $q_k^\dagger(q_\mathrm{k})$ are the creation (annihilation) operators of the rightward and leftward traveling photon modes, respectively, which follow the commutation relations $[p_k^\dagger, p_{k'}^\dagger] = \delta(k - k')$ and $[q_k^\dagger, q_{k'}^\dagger] = \delta(k - k')$. The second row of Eq. (S1) represents the interaction between the traveling photons and two magnon modes. $g_{1,2}$ are the coupling strength between magnon modes and traveling photon modes. $\Phi$ is the propagating phase between two magnon modes.

We only focus on the subsystem which is made of two magnon modes and embedded in traveling photon bath. To characterize the time evolution of this subsystem, we use Feshbach projection approach[1] to create an effective non-Hermitian Hamiltonian of the subsystem, which can be expressed in these three terms,

$$\mathcal{H}_{eff}(\omega) = H_0 + \Delta(\omega) - \frac{i}{2}\Gamma(\omega) \qquad \text{(S2)}$$

where the $H_0$ is the subspace of the $H$ and can be written in matrix form,

$$H_0 = \begin{bmatrix} \omega_1 - i\gamma_1 & 0 \\ 0 & \omega_2 - i\gamma_2 \end{bmatrix} \qquad \text{(S3)}$$

the zero off-diagonal term means that there is no direct coupling between two magnon mode. The correction term $\Delta(\omega)$ and $\Gamma(\omega)$ represent the energy shift and decay due to indirect coupling. Before obtained these two terms, we introduce the operator $A(\omega)$ to characterize the system-environmental coupling,

$$A(\omega) = \begin{bmatrix} g_1 & g_2 \mathrm{e}^{-i\Phi} \\ g_1 & g_2 \mathrm{e}^{i\Phi} \end{bmatrix} \qquad \text{(S4)}$$

the matrix element $A(\omega)_\mathrm{nm} = \langle \phi_\mathrm{n} | H | \psi_\mathrm{m} \rangle$. Where the $\psi_{\mathrm{m}=1,2}$ are the wave function of the m-th magnon mode, $\phi_{\mathrm{n}=1,2}$ are the wave function of the rightward ($\mathrm{n} = 1$) and leftward ($\mathrm{n} = 2$) traveling photon modes, respectively.

Then $\Delta(\omega)$ and $\Gamma(\omega)$ can be expressed by $A$ matrix.

$$\Delta(\omega) = \mathrm{PV}\left(\int \frac{A^\dagger(\omega')A(\omega')}{\omega - \omega'} \mathrm{d}\omega'\right) = \begin{bmatrix} 0 & \Gamma sin\Phi \\ \Gamma sin\Phi & 0 \end{bmatrix} \qquad \text{(S5)}$$

$$\Gamma(\omega) = 2\pi A^\dagger(\omega)A(\omega) = \begin{bmatrix} 2\kappa_1 & 2\Gamma cos\Phi \\ 2\Gamma cos\Phi & 2\kappa_2 \end{bmatrix} \qquad \text{(S6)}$$

where $g_{1,2} = \sqrt{\kappa_{1,2}/2\pi}$, $\Gamma = \sqrt{\kappa_1\kappa_2}$, with $\kappa_{1,2}$ are the extrinsic damping rates of two magnon modes.

The derivation of the $\Delta(\omega)$ uses the following fact,

$$\mathrm{PV}\left(\int \frac{\mathrm{e}^{i\omega' t}}{\omega-\omega'}\mathrm{d}\omega'\right) = -i\pi \mathrm{sgn}(t)\mathrm{e}^{i\omega t} \tag{S7}$$

and $\Phi \propto \omega$.

Substituting Eqs. (S5) and (S6) into Eq. (S2), we obtain the effective non-Hermitian Hamiltonian,

$$H_{eff}(\omega) = \begin{bmatrix} \omega_1 - i(\gamma_1+\kappa_1) & -i\Gamma \mathrm{e}^{i\Phi} \\ -i\Gamma \mathrm{e}^{i\Phi} & \omega_2 - i(\gamma_2+\kappa_2) \end{bmatrix} \tag{S8}$$

The $S$ matrix is given by,

$$S(\omega) = \mathrm{C}\{\mathrm{I} - 2\pi i A(\omega)\frac{1}{\omega - H_{eff}(\omega)}A^{\dagger}(\omega)\} \tag{S9}$$

where $\mathrm{C} = \begin{pmatrix} 0 & 1 \\ 1 & 0 \end{pmatrix}$ and I is identity matrix.

The matrix element of $S$ matrix is equivalent to Eqs. (S22)-(S25) that we would present below.

**(2) The classical model and spectra.** This section, we built a classical theory according to standard microwave circuit theory[2] to prove the consistency between the effective non-Hermitian Hamiltonian and ABCD matrix. Then, we derive the expression of transmission and reflection spectra in the waveguide.

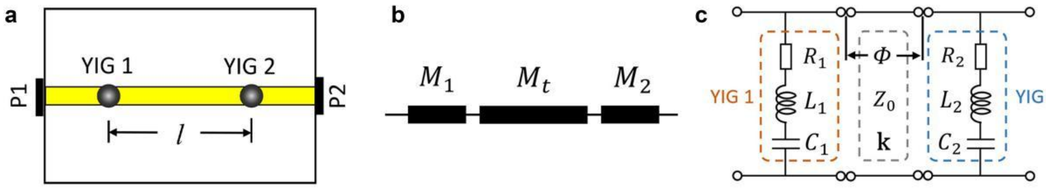


**FIG. S1 The characterization of waveguide magnonic device.** (a) Schematic diagram of the waveguide magnonic device. (b) The ideal topology of the system consists of two resonant circuits (two YIG spheres) and a transmission line, $M_1$, $M_t$, $M_2$ represent the cascade matrices of resonator 1, transmission line, and resonator 2, respectively. (c) Equivalent circuit of the coupled system.

As shown in FIG. S1 (a), two resonators (two YIG spheres) side-coupled to a transmission line and the system can be characterized by an effective RLC circuit. To describe the coupling we consider indirect interaction between two YIGs mediated by electromagnetic field of travelling wave. Two YIG spheres act as two resonant circuits with self resistance $R_{1,2}$, inductance $L_{1,2}$ and capacitance $C_{1,2}$ connected in series, the subscript 1, 2 represent YIG 1 and YIG 2, respectively, and they are connected in parallel with the transmission line contributing characteristic impedance $Z_0 = 50\ \Omega$, as well as the electrical length $\Phi = |\mathbf{k}|l = 2\pi l/\lambda$. The complex impedance of two resonant circuits can be written as $Z_1 = R_1 - i\omega L_1 - 1/i\omega C_1$ and $Z_2 = R_2 - i\omega L_2 - 1/i\omega C_2$. Because the feature dimensions of our device are much smaller than the wavelength of the microwaves, the scattering properties can be modeled by the cascade matrices $M_1$, $M_t$, $M_2$ (FIG. S1 (b)), and the ABCD matrix for the whole system can be calculated by multiplying the matrices of the individual two-port element[2],

$$\begin{bmatrix} A & B \\ C & D \end{bmatrix} = M_1 M_t M_2 = \begin{bmatrix} \cos\Phi - i\frac{Z_0}{Z_2}\sin\Phi & -iZ_0\sin\Phi \\ \left(\frac{1}{Z_1}+\frac{1}{Z_2}\right)\cos\Phi - i\left(\frac{Z_0}{Z_1 Z_2}+\frac{1}{Z_0}\right)\sin\Phi & \cos\Phi - i\frac{Z_0}{Z_1}\sin\Phi \end{bmatrix} \tag{S10}$$

where,

$$M_1 = \begin{bmatrix} 1 & 0 \\ Z_1^{-1} & 1 \end{bmatrix} \tag{S11}$$

$$M_t = \begin{bmatrix} \cos\Phi & -iZ_0\sin\Phi \\ -iZ_0^{-1}\sin\Phi & \cos\Phi \end{bmatrix} \tag{S12}$$

$$M_2 = \begin{bmatrix} 1 & 0 \\ Z_2^{-1} & 1 \end{bmatrix} \tag{S13}$$

Defining the resonant frequencies of two magnon modes as $\omega_1 = 1/\sqrt{L_1 C_1}$ and $\omega_2 = 1/\sqrt{L_2 C_2}$, the intrinsic damping rates as $\gamma_1 = R_1/2L_1$ and $\gamma_2 = R_2/2L_2$, as well as the external damping rates $\kappa_1 = Z_0/4L_1$ and $\kappa_2 = Z_0/4L_2$, according to this ABCD matrix, the transmission and reflection spectra can be derived by,

$$S_{11}(\omega) = \frac{A + B/Z_0 - CZ_0 - D}{A + B/Z_0 + CZ_0 + D} \tag{S14}$$

$$S_{21}(\omega) = \frac{2}{A + B/Z_0 + CZ_0 + D} \tag{S15}$$

$$S_{12}(\omega) = \frac{2(AD - BC)}{A + B/Z_0 + CZ_0 + D} \tag{S16}$$

$$S_{22}(\omega) = \frac{-A + B/Z_0 - CZ_0 + D}{A + B/Z_0 + CZ_0 + D} \tag{S17}$$

where $S_{11}(\omega)$ and $S_{21}(\omega)$ represent the reflection and transmission when microwaves are input form port 1 of the waveguide, $S_{22}(\omega)$ and $S_{12}(\omega)$ represent the case when microwaves are input form port 2. Substituting Eq. (S10) into Eqs. (S14)-(S17), we obtain,

$$S_{11}(\omega) =- 2i\omega \frac{\kappa_1\left[\omega^2 - \omega_2^2 + 2i\omega(\gamma_2 + \kappa_2)\right] + \kappa_2 e^{2i\Phi}\left[\omega^2 - \omega_1^2 + 2i\omega(\gamma_1 - \kappa_1)\right]}{\left[\omega^2 - \omega_1^2 + 2i\omega(\gamma_1 + \kappa_1)\right]\left[\omega^2 - \omega_2^2 + 2i\omega(\gamma_2 + \kappa_2)\right] + 4\omega^2\kappa_1\kappa_2 e^{2i\Phi}} \tag{S18}$$

$$S_{21}(\omega) = \frac{\left(\omega^2 - \omega_1^2 + 2i\omega\gamma_1\right)\left(\omega^2 - \omega_2^2 + 2i\omega\gamma_2\right)e^{i\Phi}}{\left[\omega^2 - \omega_1^2 + 2i\omega(\gamma_1 + \kappa_1)\right]\left[\omega^2 - \omega_2^2 + 2i\omega(\gamma_2 + \kappa_2)\right] + 4\omega^2\kappa_1\kappa_2 e^{2i\Phi}} \tag{S19}$$

$$S_{12}(\omega) = S_{21}(\omega) \tag{S20}$$

$$S_{22}(\omega) =- 2i\omega \frac{\kappa_1 e^{2i\Phi}\left[\omega^2 - \omega_2^2 + 2i\omega(\gamma_2 - \kappa_2)\right] + \kappa_2\left[\omega^2 - \omega_1^2 + 2i\omega(\gamma_1 + \kappa_1)\right]}{\left[\omega^2 - \omega_1^2 + 2i\omega(\gamma_1 + \kappa_1)\right]\left[\omega^2 - \omega_2^2 + 2i\omega(\gamma_2 + \kappa_2)\right] + 4\omega^2\kappa_1\kappa_2 e^{2i\Phi}} \tag{S21}$$

If we consider frequency near the coupling region, $\omega^2 - \omega_{1,2}^2 \sim 2\omega\left(\omega - \omega_{1,2}\right)$, which is the classical version of the rotating-wave approximation ignoring the term of high-frequency oscillation, then, these four S-parameters are written as,

$$S_{11}(\omega) =- i\frac{\kappa_1[\Delta_2 + i(\gamma_2 + \kappa_2)] + \kappa_2 e^{2i\Phi}[\Delta_1 + i(\gamma_1 - \kappa_1)]}{[\Delta_1 + i(\gamma_1 + \kappa_1)][\Delta_2 + i(\gamma_2 + \kappa_2)] + \kappa_1\kappa_2 e^{2i\Phi}} \tag{S22}$$

$$S_{21}(\omega)=\frac{(\Delta_1+i\gamma_1)(\Delta_2+i\gamma_2)e^{i\Phi}}{[\Delta_1+i(\gamma_1+\kappa_1)][\Delta_2+i(\gamma_2+\kappa_2)]+\kappa_1\kappa_2 e^{2i\Phi}} \tag{S23}$$

$$S_{12}(\omega)=S_{21}(\omega) \tag{S24}$$

$$S_{22}(\omega)=-i\frac{\kappa_1 e^{2i\Phi}[\Delta_2+i(\gamma_2-\kappa_2)]+\kappa_2[\Delta_1+i(\gamma_1+\kappa_1)]}{[\Delta_1+i(\gamma_1+\kappa_1)][\Delta_2+i(\gamma_2+\kappa_2)]+\kappa_1\kappa_2 e^{2i\Phi}} \tag{S25}$$

where $\Delta_1=\omega-\omega_1$ and $\Delta_2=\omega-\omega_2$ are the frequency detunings of two magnon modes.

It's worth to noting that, the poles of these S-parameters in complex frequency plane, or zero denominator in Eqs. (S22)-(S25), correspond to the eigenvalues of Eq. (S8). That proves the consistency between the classical theory and the effective non-Hermitian Hamiltonian we discussed above.

**(3) Fabry-Pérot (FP)-like resonant mechanism.** As shown in FIG. S2, considering microwaves are input from the left side of resonator *a*, and the multiple reflection between two resonators.

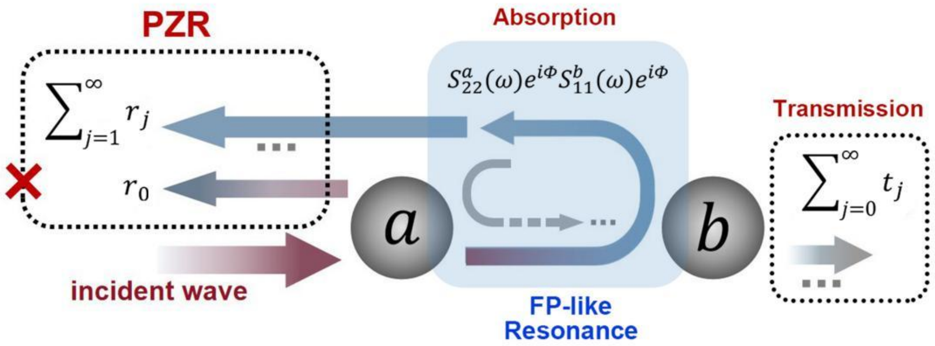


**FIG. S2 Schematic diagram of wave propagation in two-magnon system.**

For the isolated mode *a*, the reflection and transmission are in the forms,

$$S^a_{11}(\omega)=S^a_{22}(\omega)=-\frac{2i\omega\kappa_1}{\omega^2-\omega_1^2+2i\omega(\gamma_1+\kappa_1)} \tag{S26}$$

$$S^a_{21}(\omega)=S^a_{12}(\omega)=1-\frac{2i\omega\kappa_1}{\omega^2-\omega_1^2+2i\omega(\gamma_1+\kappa_1)} \tag{S27}$$

For the isolated mode *b*, the reflection and transmission are in the forms,

$$S^b_{11}(\omega)=S^b_{22}(\omega)=-\frac{2i\omega\kappa_2}{\omega^2-\omega_2^2+2i\omega(\gamma_2+\kappa_2)} \tag{S28}$$

$$S^b_{21}(\omega)=S^b_{12}(\omega)=1-\frac{2i\omega\kappa_2}{\omega^2-\omega_2^2+2i\omega(\gamma_2+\kappa_2)} \tag{S29}$$

Considering the round-way behavior between two modes (interaction loop between modes *a* and *b* as shown in FIG. S2) and defining,

$$w=S^a_{22}(\omega)e^{i\Phi}S^b_{11}(\omega)e^{i\Phi} \tag{S30}$$

Substituting Eqs. (S26) and (S28) into Eq. (S30), it can be written as,

$$w=\frac{-2i\omega\kappa_1}{\omega^2-\omega_1^2+2i\omega(\gamma_1+\kappa_1)}\frac{-2i\omega\kappa_2}{\omega^2-\omega_2^2+2i\omega(\gamma_2+\kappa_2)}e^{2i\Phi} \tag{S31}$$

Setting $w\equiv 1$ in Eq. (S31) and taking the approximation $\omega^2-\omega_1^2\sim 2\omega(\omega-\omega_1)$ and $\omega^2-\omega_2^2\sim 2\omega(\omega-\omega_2)$ (near the coupling region), we obtain,

$$\left[\frac{i\kappa_1}{\omega-\omega_1+i(\gamma_1+\kappa_1)}\right]\left[\frac{i\kappa_2}{\omega-\omega_2+i(\gamma_2+\kappa_2)}\right]e^{2i\Phi}=1 \tag{S32}$$

This is exactly the eigen-equation as follow,

$$[\omega-\omega_1+i(\gamma_1+\kappa_1)][\omega-\omega_2+i(\gamma_2+\kappa_2)]+\kappa_1\kappa_2 e^{2i\Phi}=0 \tag{S33}$$

The eigenvalues ($\widetilde{\omega}_{\pm}$) in Eq. (2) of the main text is the solution of above eigen-equation, which is also consistent with the theory we discussed in Supplementary Note 1 (1) and (2).

**Supplementary Note 2. Zero-reflection condition and perfect zero-reflection condition**

**(1) Zero-reflection (ZR) condition.** Setting $S_{11}(\omega)$ =0 and $S_{22}(\omega)$ =0 in Eqs. (S18) and (S21), we derive the zero-reflection (ZR) conditions as follow. For the case that microwaves are input from port 1,

$$\widetilde{\omega}_{ZR}^{1}=\frac{-i\zeta_1+\sqrt{(\kappa_1+\kappa_2 e^{2i\Phi})(\kappa_2\omega_1^2 e^{2i\Phi}+\kappa_1\omega_2^2)-\zeta_1^2}}{\kappa_1+\kappa_2 e^{2i\Phi}} \tag{S34}$$

where $\zeta_1=\kappa_1(\gamma_2+\kappa_2)+\kappa_2(\gamma_1-\kappa_1)e^{2i\Phi}$ . Here, the other solution of Eq. (S34) has been rejected due to the the limitation of parameter space in our system.

For for the case that microwaves are input from port 2,

$$\widetilde{\omega}_{ZR}^{2}=\frac{-i\zeta_2+\sqrt{(\kappa_1 e^{2i\Phi}+\kappa_2)(\kappa_2\omega_1^2+\kappa_1\omega_2^2 e^{2i\Phi})-\zeta_2^2}}{\kappa_1 e^{2i\Phi}+\kappa_2} \tag{S35}$$

where $\zeta_2=\kappa_1(\gamma_2-\kappa_2)e^{2i\Phi}+\kappa_2(\gamma_1+\kappa_1)$, and the other solution of Eq. (S35) has been rejected due to the the limitation of parameter space in our system. In the main text, the orange lines in Fig. 2 represent the real parts $\mathrm{Re}\,(\widetilde{\omega}_{ZR})$ and the imaginary parts $\mathrm{Im}\,(\widetilde{\omega}_{ZR})$ of the ZR conditions, which are numerically calculated from Eq. (S34).

**(2) Perfect zero-reflection (PZR) condition.** Supposing the imaginary parts of ZR solutions are equal to zero $\mathrm{Im}\,(\widetilde{\omega}_{ZR})=0$ , from Eqs. (S34) and (S35), the PZR condition is derived as,

$$\omega_{PZR}^{\pm}=\frac{1}{2}\frac{sin\,(2\Phi)\,(\omega_1^2-\omega_2^2)}{(\kappa_2-\kappa_1)(cos\,2\,\Phi-1)\pm\left[(\gamma_1+\gamma_2)\,cos\,(2\Phi)+\frac{\kappa_1\gamma_2}{\kappa_2}+\frac{\kappa_2\gamma_1}{\kappa_1}\right]} \tag{S36a}$$

the superscript "$\pm$" represents the case that microwaves are input from port 1 and from port 2, respectively. Notably, $\omega_1$ and $\omega_2$ in Eq. (S36a) should satisfy:

$$(\omega_1^2-\omega_2^2)^2/(\mathcal{C}'\omega_1^2-\mathcal{C}\omega_2^2)=4(\mathcal{C}'-\mathcal{C}) \tag{S36b}$$

where $\mathcal{C}=[\kappa_2(\kappa_1-\gamma_1)cos2\Phi-\kappa_1(\gamma_2+\gamma_2)]/\kappa_2 sin2\Phi$ and $\mathcal{C}'=[-\kappa_2(\kappa_1-\gamma_1)-\kappa_1(\gamma_2+\kappa_2)cos2\Phi]/\kappa_1 sin2\Phi$.

Here, we also provide a more intuitive physical picture and mathematical analysis to understand the PZR. As shown in FIG. S2, defining the *j*-th reflection at mode *a* as $r_j$, therefore,

$$r_0=S_{11}^a(\omega)$$

$$r_1=S_{21}^a(\omega)e^{i\Phi}S_{11}^b(\omega)e^{i\Phi}S_{12}^a(\omega)$$

$$r_2=S_{21}^a(\omega)e^{i\Phi}S_{11}^b(\omega)e^{i\Phi}S_{22}^a(\omega)e^{i\Phi}S_{11}^b(\omega)e^{i\Phi}S_{12}^a(\omega)$$

...

$$\sum_{j=1}^{\infty}r_j=\frac{S_{21}^a(\omega)e^{i\Phi}S_{11}^b(\omega)e^{i\Phi}S_{12}^a(\omega)}{1-S_{22}^a(\omega)e^{i\Phi}S_{11}^b(\omega)e^{i\Phi}} \tag{S37}$$

If we substitute the experimental parameters of PZR into $r_0$ and $\sum_{j=1}^{\infty}r_j$ above, the real part and

imaginary part of $\sum_{j=1}^{\infty} r_j$ would cancel that in $r_0$. That indicates the destructive interference of multiple reflected waves results in the PZR.

**Supplementary Note 3. Experimental spectra of transmission, reflection and absorption**

Defining the reflectivity and transmittivity as $r_1 = |S_{11}|^2$ and $t_1 = |S_{21}|^2$, the absorptivity of the system can be given by $A_1 = 1 - r_1 - t_1$. In experiment, the propagating delay phase $\Phi$ is continuously regulated by applying different external magnetic field (**H**-field). Three typical **H**-fields are fixed at $\mu_0|\mathbf{H}| = 1160$ Gs, 1380 Gs, 1524 Gs, which determine $\Phi = 1.07\pi$ ($\omega_2/2\pi = 3.6$ GHz), $\Phi = 1.25\pi$ ($\omega_2/2\pi = 4.24$ GHz) and $\Phi = 1.37\pi$ ($\omega_2/2\pi = 4.69$ GHz), respectively. In each case of $\Phi$, sweeping coil current under the YIG 1 to adjust $\omega_1$, we obtain the reflectivity $r_1$ (the 1st row), transmittivity $t_1$ (the 2nd row) and absorptivity $A_1$ (the 3rd row) mappings as functions of $\Delta_H = \omega_1 - \omega_2$ and $\Delta_2 = \omega - \omega_2$, as shown in FIG. S3, each column corresponds to different cases of $\Phi$. Black Dashed curves in FIGs. S3 (a)-(c) are the calculated eigenvalues from Eq. (2) in the main text with parameters $\gamma_1/2\pi = \gamma_2/2\pi = 1$ MHz, $\kappa_1/2\pi = 1.7$ MHz and $\kappa_2/2\pi = 3.3$ MHz. Obviously, the dispersions of the hybridized modes transit from level attraction to level repulsion by non-Hermitian control ($\Phi$). Red lines are the ZR conditions calculated by Eq. (S34). Circles mark the perfect zero-reflection (PZR) conditions that have infinitely narrow linewidth in measured reflection spectra, and these conditions agree well with the prediction of Eq. (S36). When the PZR condition approach to the hybridized mode, as shown in FIG. S3 (b), the nearly full absorption (NFA) can be realized. Without loss of generality, the measured mappings of reflectivity $r_2$, transmissivity $t_2$ and absorption $A_2$ are displayed in FIG. S4.

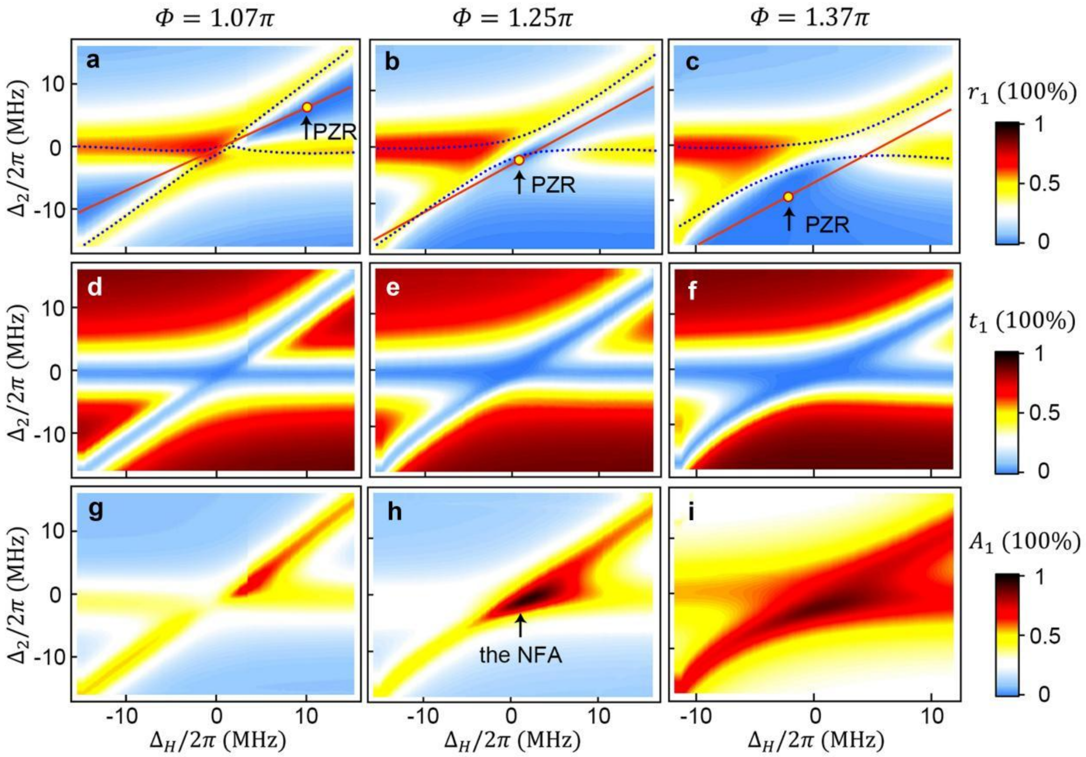


**FIG. S3 The experimental mappings of reflectivity, transmittivity and absorptivity for the**

**case that microwaves are input from port 1.** Mappings of (a)-(c) are reflectivity $r_1$, (d)-(f) transmissivity $t_1$ and (g)-(i) absorptivity ($A_1$) spectra as functions of $\Delta_H$ and $\Delta_2$ in three cases: $\Phi = 1.07\pi, 1.25\pi, 1.37\pi$ , respectively. In (a)-(c), black dashed curves are the calculated eigenvalues of the hybridized modes $\Delta\mathrm{Re}(\widetilde{\omega}_\pm) = \mathrm{Re}(\widetilde{\omega}_\pm) - \omega_2$ from Eq. (2), red lines are the calculated ZR conditions from Eq. (S34), circles represent the PZR conditions observed in experiment. When $\Phi = 1.25\pi$, the nearly full absorption (NFA) is realized at the PZR frequency as arrow marked in (h).

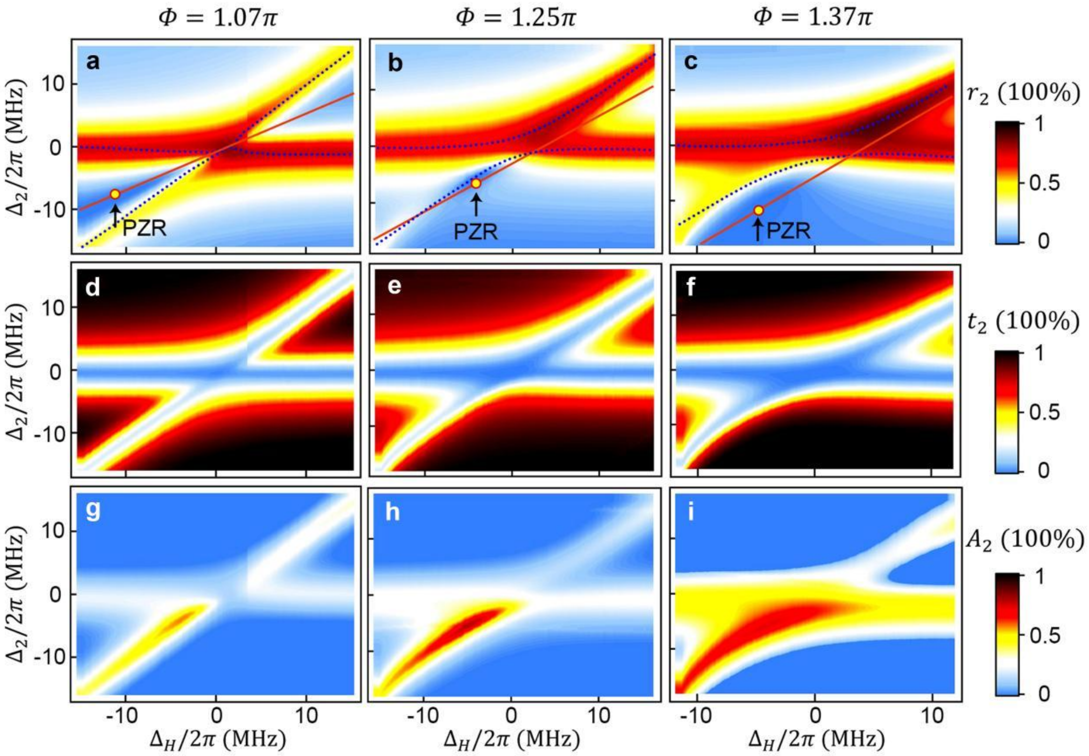


**FIG. S4 The experimental mappings of reflectivity, transmittivity and absorptivity for the case that microwaves are input from port 2.** Mappings of (a)-(c) are reflectivity $r_2$, (d)-(f) transmissivity $t_2$ and (g)-(i) absorptivity ($A_2$) spectra as functions of $\Delta_H$ and $\Delta_2$ in three cases: $\Phi = 1.07\pi, 1.25\pi, 1.37\pi$ , respectively. In (a)-(c), black dashed curves are the calculated eigenvalues of the hybridized modes $\Delta\mathrm{Re}(\widetilde{\omega}_\pm) = \mathrm{Re}(\widetilde{\omega}_\pm) - \omega_2$ from Eq. (2), red lines are the calculated ZR conditions from Eq. (S35), circles represent the PZR conditions observed in experiment.

FIGs. S5 (a)-(c) show the transmissivity $t_1$ (blue curve) and absorption $A_1$ (red curve) spectra extracted at the PZR conditions (arrows marked field in FIG. S3). Without loss of generality, the measured transmissivity $t_2$ and absorption $A_2$ spectra at the PZR conditions are given in FIGs. S5 (d)-(f). The PZR spectra $|S_{11}|$ and $|S_{22}|$ with unit of dB (corresponding to the right scale) are plotted as gray curves for guiding to eyes, these spectra show ultra-sharp dips with infinitely narrow linewidth. At the minimal amplitude, $|S_{11(22)}| = 0$ , we mark the measured transmitivity/absorptivity values by blue diamond/red triangle, which are good accord with the theoretical prediction of Figs. 4a, b in the main text.

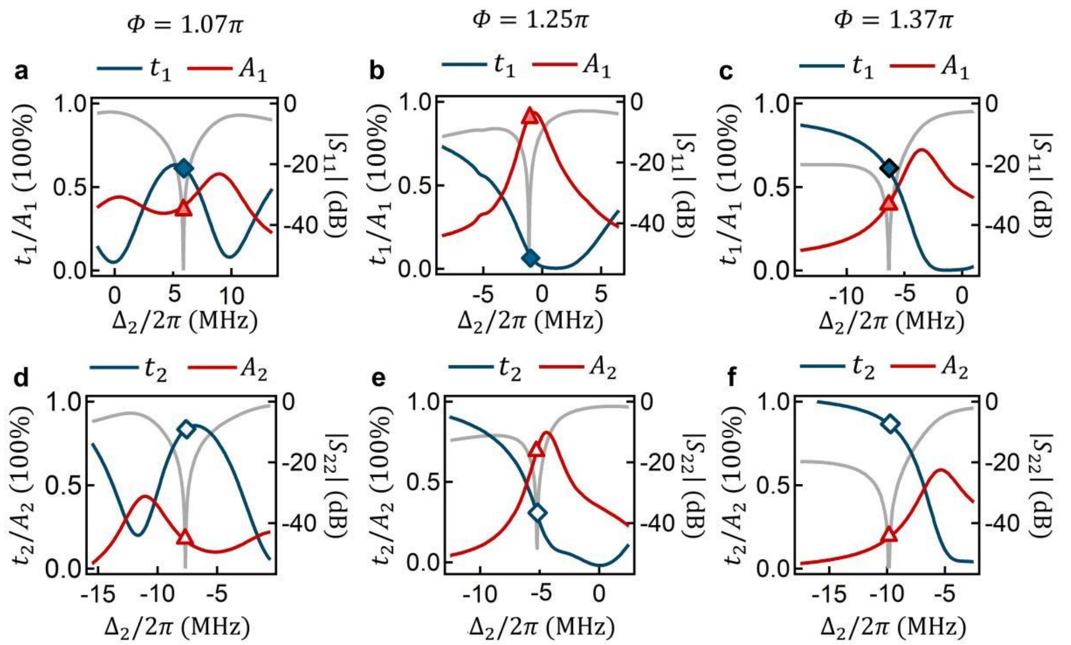


**FIG. S5 The measured spectra under the PZR conditon.** When microwaves are input from port 1, the 1st row displays the measured spectra of transmittivity $t_1$ (blue curve) and absorptive $A_1$ (red curve) at the PZR conditions in three different cases: (a) $\Phi = 1.07\pi$, (b) $\Phi = 1.25\pi$, and (c) $\Phi = 1.37\pi$. When microwaves are input from port 2, the 2nd row displays the measured spectra of transmittivity $t_2$ (blue curve) and absorptive $A_2$ (red curve) at the PZR condition in three different cases: (d) $\Phi = 1.07\pi$, (e) $\Phi = 1.25\pi$, and (f) $\Phi = 1.37\pi$. The PZR spectra $|S_{11(22)}|$ corresponding to the right scale are plotted by gray curves for guiding to eyes.

### Supplementary Note 4. Discussion about the maximum energy absorption

Considering two magnon modes have identical intrinsic damping rates $\gamma_1/2\pi = \gamma_2/2\pi = 1$ MHz, we discuss how does the dissipation ratio $\kappa_2/\kappa_1$ effect the maximum absorption.

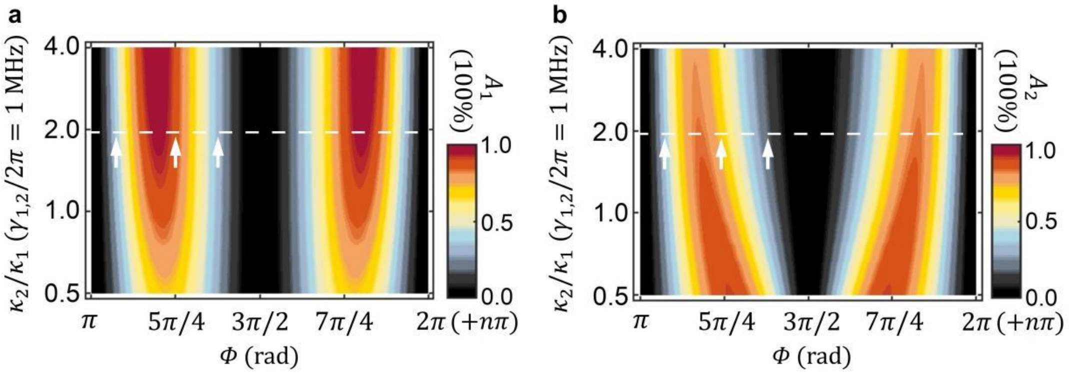


**FIG. S6 The calculated maximum absorptivity.** (a) $A_1$ and (b) $A_2$ as functions of $\Phi$ and $\kappa_2/\kappa_1$ under the PZR. In calculations, $\gamma_1/2\pi = \gamma_2/2\pi = 1$ MHz, $\kappa_1/2\pi = 1.7$ MHz, $\kappa_2/2\pi = 3.3$ MHz are set based on the fitting results from experimental spectra. The dissipation ratio $\kappa_2/\kappa_1$ of our configuration is set on the dashed lines represented condition. All measurements displayed in this work are carried out under three $\Phi$ values as arrows marked in (a) and (b).

In the premise of PZR condition Eq. (S36), we calculate the maximum absorption as a function of $\Phi$ and $\kappa_2/\kappa_1$, as shown in FIG. S6. For microwaves are input from port 1 (FIG. S6 (a)), nearly full absorption (NFA) ($A_1 > 90\%$) can be realized as long as $\kappa_2/\kappa_1 > 1.5$, and $A_1$ can be further increased by improving the ratio of $\kappa_2/\kappa_1$. While for microwaves input from port 2 (Fig. S6 (b)), $A_2 > 90\%$ appears when $\kappa_2/\kappa_1$ <0.6. These results indicate that, to achieve the nearly full absorption (NFA), we should enable the dissipation of two YIG spheres is asymmetry, and the extrinsic damping rate of which close to the output port should be larger than which one close to the input port. In our experiment, practical parameters are fixed at $\kappa_2/\kappa_1 = 1.94$ as dashed line marked in FIG. S6, the arrows marked values $\Phi = 1.07\pi$, $1.25\pi$, $1.37\pi$ are three conditions when we measure the mappings of spectra.

**Supplementary References**


[1] Ashida, Y., Gong, Z. & Ueda, M. Non-hermitian physics. Adv. Phys. **69**, 249-435 (2020).

[2] Pozar, D. M. Microwave engineering. John wiley & sons, 2009.
